# ACCELEROMETRY-BASED PHYSICAL ACTIVITY (MIMS/DAY) AND FALL RISK APPRAISAL MATRIX IN OLDER ADULTS

**DOI:** 10.1093/geroni/igad104.2271

**Published:** 2023-12-21

**Authors:** Renoa Choudhury, Joon-Hyuk Park, Chitra Banarjee, David Fukuda, Rui Xie, Jeffrey Stout, Ladda Thiamwong

**Affiliations:** University of Central Florida, Orlando, Florida, United States; University of Central Florida, Orlando, Florida, United States; University of Central Florida, Orlando, Florida, United States; University of Central Florida, Orlando, Florida, United States; University of Central Florida, Orlando, Florida, United States; University of Central Florida, Orlando, Florida, United States; University of Central Florida, Orlando, Florida, United States

## Abstract

Fall Risk Appraisal Matrix (FRAM) is vital for understanding the discrepancy between perceived fall risk (i.e., Fear of Falling, FOF) and physiological fall risk (i.e., balance performance) in older adults. However, little is known about how habitual physical activity (PA) levels, expressed in Monitor Independent Movement Summary (MIMS) units, differ across FRAM categories. This cross-sectional study examines the associations of FRAM and wrist-worn accelerometer-based daily PA in community-dwelling older adults. FOF (Short FES-I scale), balance performance (BTrackS Balance) and PA data (ActiGraph GT9X) were collected from 156 older adults (Mean age = 76.74±9.62 years). FRAM categorizes the participants into four groups: i) Rational (low FOF-normal balance), ii) Incongruent (low FOF-poor balance), iii) Irrational (high FOF-normal balance), and iv) Congruent (high FOF-poor balance). PA data were summarized as MIMS/day (the average of daily MIMS) and peak 30-minute MIMS (the average of highest 30 non-consecutive MIMS minutes/day, representing a PA index of higher intensity epochs). Differences across groups were examined using one-way analysis of variance (ANOVA). Results indicate that, total accumulated PA (i.e., average daily MIMS) was 17% lower in Congruent FRA group than Rational FRA group (p=.02). The peak PA effort (i.e., peak 30-minute MIMS) decreased in Incongruent (9%, p=.027), Irrational (12%, p=.002) and Congruent (16%, p=.003) groups as compared to the Rational FRA group. Our results suggest that fall intervention strategies should integrate both cognitive behavioral therapy (for reducing FOF) and balance exercises to promote high-intensity PA in older adults.

